# Relationship between Bone Mineral Density and Dental Caries in Koreans by Sex and Menopausal State

**DOI:** 10.3390/ijerph19116917

**Published:** 2022-06-05

**Authors:** Yun-Hee Lee, Jun-Pyo Myong

**Affiliations:** 1 Department of Urology, College of Medicine, The Catholic University of Korea, 222, Banpo-daero, Seocho-gu, Seoul 06591, Korea; eyh900@catholic.ac.kr; 2 Department of Occupational & Environmental Medicine, Seoul St. Mary’s Hospital, College of Medicine, The Catholic University of Korea, 222, Banpo-daero, Seocho-gu, Seoul 06591, Korea

**Keywords:** bone density, dental caries, osteoporosis, decayed, missing, and filled teeth index

## Abstract

We aimed to investigate the relationship between bone mineral density and dental caries in adults of over 19 years of age who were categorized according to their sex and menopausal status. The Korea National Health and Nutrition Examination Survey (KNHANES) dataset was used for the study. Bone mineral density (BMD) and oral health examination data were collected between 2008 and 2011. A total of 17,141 adults of ≥19 years old were eligible for inclusion in the present study. Multiple regression analysis was performed after adjustment for age, household income, educational level, smoking status, and alcohol drinking status for men, and pre- and post-menopausal women. In men, the β-value for the mean decayed, missing, and filled teeth (DMFT) index was 0.98 (95% confidence interval (CI) = 0.71–1.25), and was significantly higher in osteoporotic participants than in participants with normal BMD (*p* < 0.05). In post-menopausal women, the β-value for the mean DMFT index was 0.86, and was higher in the osteoporotic participants than in the participants with normal BMD (*p* < 0.05). Men and post-menopausal women with osteoporosis had higher DMFT indexes than those with normal BMD. In addition, there was a correlation between DMFT index and BMD in men and post-menopausal women. Therefore, the prevention of osteoporosis should be implemented alongside proper oral care.

## 1. Introduction

Osteoporosis is the best-known bone disease and is associated with increases in incidences of fracture and mortality. In particular, post-menopausal women commonly develop osteoporosis because of a marked decrease in estrogen level [1], and it is closely associated with poor quality of life [2]. Although osteoporosis is less common in men than in women, it is an important problem in both sexes worldwide, and more effective preventive measures are needed to reduce the cost of medical care [3,4].

Dental caries is caused by carbohydrates. Excessive intake of carbohydrates causes enamel to decay, resulting in dental caries, which can lead to tooth loss [5]. Dental caries has a high prevalence worldwide, and is a disease requiring management [6]. It is widely seen not only in children, but also in older adults [7].

Bone health and oral health are connected because a reduction in bone mineral density (BMD) is associated with a decrease in alveolar bone height [8], and BMD also affects the progression of increases to the absent decayed, missing, and filled teeth (DMFT) index and its sequelae, such as dental caries, periodontal disease, tooth mobility, and tooth loss [9,10].

Although the results of several studies of the relationship between BMD and oral disease in post-menopausal women have been reported, their findings have been inconsistent [11,12], and until now, many studies on the relationship between periodontal disease and BMD have been published. However, compared to other oral diseases, the relationship between dental caries and BMD is insufficiently studied.

Relatively recently, papers have also been published that explain the relationship between bone health and dental caries [13], or analyze the significant correlation between BMD and dental caries through T-score and DMFT index [14,15].

The present study was conducted to investigate the relationship between BMD and dental caries in adults of over 19 years of age who were classified as men, pre-menopausal women, or post-menopausal women, using data from the Korea National Health and Nutrition Examination Survey (KNHANES). One thing to consider is that both age and endocrine status are likely to affect the relationship between dental caries and bone health. Therefore, it is important to control for the effects of age, endocrine status, and sex when evaluating this relationship.

## 2. Materials and Methods

### 2.1. Materials

The KNHANES dataset was used for the present study. The participants in KNHANES were selected using stratified, multi-stage, clustered probability methods to provide a sample of the non-institutionalized Korean general population. This survey was conducted with the approval of the Research Ethics Review Committee of the Korea Centers for Disease Control (KCDC) and Prevention. Data were collected between 2008 and 2011 (KNHANES IV and V), which included information regarding BMD and the findings of oral health examinations. The participation rates were 77.8% in 2008, 82.8% in 2009, 81.9% in 2010, and 80.0% in 2011. Although more recent KNHANES data exist, the reason for using historical data is that bone mineral density tests were performed only from 2008 to 2011.

### 2.2. Study Population

**Figure 1:**
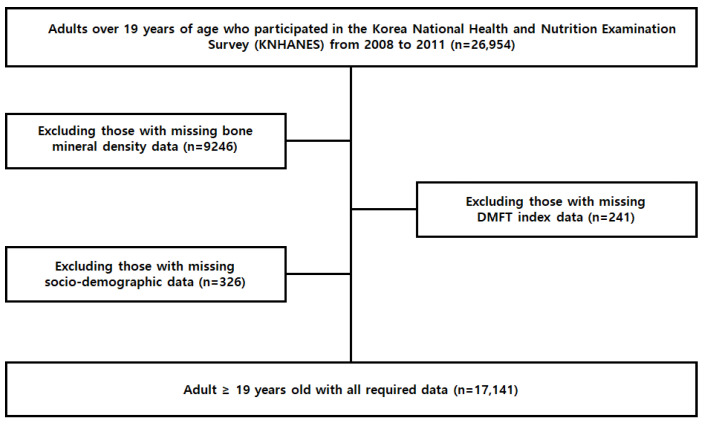
Flow chart indicating the subset of participants included for the analysis from the NHANES 2008–2011 dataset.

The inclusion criteria were selected by using the survey area of the Statistics Korea (KOSTAT) Population and Housing Census as an extraction frame, and household members over the age of 1 in the selected households were surveyed. There was a total of 35,913 participants in KNHANES between 2008 and 2011. The exclusion criteria were as follows: absent BMD data (n = 9487), <19 years of age (n = 8959), DMFT index (n = 241), and absent socio-demographic data (n = 85). The final study sample consisted of 17,141 men and women aged ≥19 years for whom BMD, DMFT index, and socio-demographic data were available (Figure 1).

### 2.3. Variables

Participant sex was classified as follows: male, pre-menopausal female, or post-menopausal female, according to the response to a question regarding the experience of menopause. Age was classified as 19–29 years, 30–39 years, 40–49 years, 50–59 years, 60–69 years, or ≥70 years. Menopause was assessed by yes, no, hysterectomy, and not applicable (men).

BMD data were collected for the entire femur, the femoral neck, and the lumbar spine. BMD was measured using an X-ray BMD meter (Discovery-W fan-beam densitometer, Hologic, Inc., Bedford, MA) by dual-energy X-ray absorptiometry. Although the participants’ X-ray exposure was inevitable, this investigation was conducted within the permitted range after obtaining approval for research ethics in advance. For quality control purposes, each investigator measured BMDs (g/cm^2^), and sent the results to the quality control team, and the lead investigator interpreted the results of BMD analyses every week with the responsible researcher. At this time, the interpretation was carried out according to the BMD test result interpretation guidelines. If there was a discrepancy, the researcher informed the investigator and requested re-analysis if necessary. In addition, the researchers were evaluated for the precision of their estimates and their performance in training, and their technique was corrected if required. There was no national standard for the calculation of the T-score, which is an index of the degree of osteoporosis; therefore, this was calculated on the basis of the maximum BMD in Asia (Japan) [16]. The criteria used for the diagnosis of osteoporosis were as defined by the World Health Organization (WHO): T-score ≥ −1.0 was regarded as normal, −2.5 < T-score < −1.0 was regarded as osteopenia, and T-score ≤ 2.5 was regarded as osteoporosis [17,18]. In the present study, if the T-score for any of the entire femur, femoral neck, or lumbar spine was consistent with osteoporosis, the participant was defined as having osteoporosis. Likewise, if any one of the sites had a T-score that corresponded to osteopenia, the participant was defined as having osteopenia. Otherwise, the participant was recorded as having normal BMD.

The DMFT index is a standard means of assessing the experience of dental caries. In KNHANES, the DMFT index of each participant was assessed annually through oral examination, and it is a discrete variable with values of between 0 and 28, corresponding to the possible number of teeth. The DMFT index consists of the following factors: decayed tooth (DT), which represents caries in an untreated permanent tooth that is observed during oral examination; missing tooth (MT), which represents the permanent loss of a tooth due to dental caries; and filled tooth (FT), which represents the treatment of a permanent tooth for caries in the past. The DMFT index is the sum of the number of permanent teeth with caries, the number of permanent teeth lost due to caries, and the number of permanent teeth treated for caries. Thus, the higher the index, the more experienced the participant’s tooth decay, missing, and filling.

For household income, the monthly average household equalized income calculated by household monthly income/number of household members was classified into quartiles according to a year-by-year basis. Household income was categorized as Q1 (lowest income), Q2 (lower-middle income), Q3 (upper-middle income), and Q4 (highest income). Educational level was categorized as below elementary school graduation, middle school graduation, high school graduation, or university graduation or above. Smoking status was defined as never a smoker, or a former or current smoker based on the current smoking status question. Alcohol status was classified as a person who drank alcohol or never drank alcohol based on the question of whether or not they experienced drinking in their lifetime.

In addition to that, we added several variables related to oral cavities that can affect the DMFT index. The periodontal disease variable was defined as ‘yes’ when the Community Periodontal Index (CPI) was 3–4 mm or more at any one of the six tooth areas. Six tooth areas were categorized as upper-right molar, upper anterior, upper-left molar, lower-right molar, lower anterior, and lower-left molar. This does not include past periodontal disease experience, only the results of the current oral examination. Frequency of toothbrushing per day was observed from a minimum of 0 to a maximum of 4 after breakfast, after lunch, after dinner, and before bedtime. Necessity of oral treatment was defined as ‘yes’ if more than 1 of 28 teeth needed treatment at the time of examination, and ‘no’ otherwise. Targets defined as ‘yes’ include all cases that require simple conservative treatment, artificial crown, pulp treatment, tooth extraction, etc. The diabetes variable was marked as ‘yes’ when a doctor diagnosed diabetes for a lifetime. Any cases that had never been diagnosed or had no illness or could not remember were all defined as ‘no’.

### 2.4. Statistical Analysis

The data were analyzed using SAS version 9.4 (SAS Institute Inc., Cary, NC, USA). The general characteristics of the participants are expressed as frequencies, or means and standard errors (SEs). To increase the representativeness of the population, weighting variables were used for statistical analysis. The Rao–Scott chi-squared test was used to compare sets of categorical data. Survey regression analysis was used to determine the relationships between discrete variables, such as the DMFT index and BMD in men, and pre- and post-menopausal women. Multiple survey regression analysis was performed to evaluate the relationship between BMD and DMFT after adjustment for socioeconomic variables, such as age, household income, and educational level; and behaviors affecting health, such as smoking and alcohol drinking; and the *p*-value for the trend was calculated (level of significance <0.05).

## 3. Results

The mean DMFT indexes in the various categories of participants are shown in Table 1. The men who had the highest DMFT index of 10.1 were in the >70-year-old group; the pre-menopausal women with the highest index of 6.8 were in the 19–29-year-old group and 50–59-year-old group; and the post-menopausal women had the highest value of 11.5 in the >70-year-old group (*p* < 0.05). With regard to educational status, men and post-menopausal women had the highest DMFT indexes if they had not graduated from elementary school, but for pre-menopausal women, this was highest in university graduates. With regard to household income, men and post-menopausal women had the highest mean DMFT index in Q1, and pre-menopausal women had the highest index in Q4.

**Figure 2:**
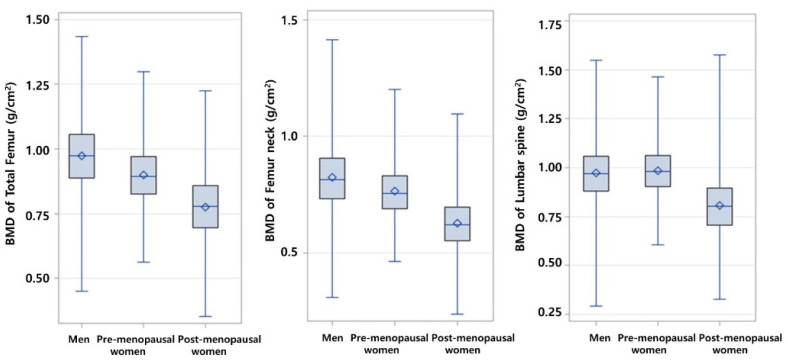
BMD by site (total femur, femur neck, lumbar spine) for male, and pre- and postmenopausal females.

Figure 2 shows the distribution of BMD by site for men, and pre- and post-menopausal women. The mean BMDs of the entire femur and femoral neck were highest in men, but that of the lumbar spine was highest in pre-menopausal women.

Table 2 shows the relationships between the mean DMFT index and BMD-related parameters in men, and pre- and post-menopausal women. In men and post-menopausal women, the mean DMFT index was higher in participants with osteoporosis than in those with normal BMD (*p* < 0.05). The proportion of participants that had osteoporosis was highest in post-menopausal women (37.8%), followed by men (4.8%) and pre-menopausal women (1.4%).

In addition to the BMD-related parameters, a number of other factors that might affect the DMFT index were selected, and multiple survey regression analysis was performed that corrected for age, household income, educational level, smoking status, alcohol drinking status, periodontal disease, frequency of toothbrushing per day, necessity of oral treatment, and the presence of diabetes (Table 3).

In men, the β-value for the mean DMFT index was 0.98 (95% confidence interval = 0.71–1.215, and this was significantly higher in participants with osteoporosis than in those with normal BMD (*p* < 0.05)). Among the men, those >70 years of age had the highest DMFT index, as did those who had not graduated from elementary school and those who were current smokers. Among the pre-menopausal women, BMD was not significantly associated with any of the other parameters. Among the post-menopausal women, the β-value for the mean DMFT index was 0.86, and this was higher in participants with osteoporosis than in those with normal BMD (*p* < 0.05). The DMFT index increased significantly with age. The women who had not graduated from elementary school and those who were current or past smokers had higher DMFT indexes than those with other educational levels and non-smokers (*p* < 0.05). On the other hand, as for the periodontal disease variable that can cause the DMFT index increase, ‘no’ has a higher DMFT index than ‘yes’. It was also confirmed that the frequency of toothbrushing per day also did not significantly affect the DMFT index decrease, even if the number of times increased. As for the necessity of oral treatment variable, subjects who needed more than one oral treatment had a significantly higher DMFT index than those who did not. These results were the same in all sex types, and were high in the order of men > pre-menopausal women > post-menopausal women.

For the presence of diabetes, the ‘yes’ group in men and pre-menopausal women had a significantly higher DMFT index than the ‘no’ group, but not in post-menopausal women.

## 4. Discussion

Men and post-menopausal women with osteoporosis had higher DMFT indexes than those with normal BMD in the present study. In addition, there was a correlation between DMFT index and BMD in both men and post-menopausal women.

There are several possible mechanisms that might explain the association between bone health and DMFT indexes. First, dental caries is a multifactorial disease, one of the causes of which is the degree of hard tissue resistance (enamel and dentin). It is a parameter related to BMD (AD-SoS value) [9]. Moreover, previous studies that analyzed that the T-score, which is a measure of BMD, and the DMFT index showed a significant correlation, which supports this argument [14,15].

Second, poor oral health, with a high DMFT index, might be associated with low food intake, which might result in low calcium levels or hormonal imbalances. Patients with oral disease may experience pain when eating [19], and such pain during chewing may limit the consumption of foods that are high in calcium [20]. Therefore, when calcium intake, which is critical for bone homeostasis, is limited, bone health may deteriorate [21].

Third, age may influence both bone health and DMFT index in men and post-menopausal women. Osteoporosis is an age-related disease [22], and old age is also associated with a high DMFT index [23]. To control for the potential confounding effect of age on both bone health and the DMFT index, the participants were stratified according to age (<50 or ≥50 years in men, <60 or ≥60 years in women; Supplementary Table S1). In the men with osteoporosis, the regression coefficients for the relationship between the DMFT index and BM\D for those aged <50 years and ≥50 years were 0.61 (*p* < 0.05) and 1.94 (*p* < 0.05), respectively. The closer relationship between BMD and the DMFT index in men aged ≥50 years was also present in post-menopausal women aged <60 years. Thus, age per se was an effect modifier, but was not a confounding factor. Therefore, a relationship between BMD and the DMFT index was shown even in the younger age groups.

Estrogen levels confounded the association between BMD and the DMFT index in the present study. After stratification of the sample according to sex and menopausal status to control for the effect of hormonal status, there was no significant relationship in pre-menopausal women in the present study. This can be explained by the protective role that estrogen has in bones in pre-menopausal women [24]. It is also associated with an increase in oral disease in post-menopausal women due to decreased estrogen [25].

Smoking status was associated with the DMFT index in men and post-menopausal women, but not in pre-menopausal women. Smoking reduces saliva secretion and causes a dry mouth, which predisposes toward oral diseases, including dental caries [26]. In addition, Streptococcus mutans, a bacterium that causes dental caries, is present in larger numbers in the saliva of smokers than in non-smokers [27]. According to Myong et al. (2013), the sensitivities of self-reported smoking status were only 46.6% and 47.5% in Korean pre- and post-menopausal women, respectively [28]. The Confucian culture might cause women to keep their smoking secret, and this might have influenced the relationship between smoking status and DMFT in women. In fact, Korean females often hide their smoking status, so the smoking rate is reported to be low [29].

The present study had some limitations. First, since the DMFT index represents a lifetime experience, the experience of dental caries in childhood cannot be excluded. Because osteoporosis occurs mainly after adulthood, dental caries suffered in childhood are a limitation when explaining the correlation between BMD and the DMFT index. However, the DMFT index is a widely-used indicator of general oral health in epidemiological studies, and the WHO recommends its use to assess and compare the effects of dental caries [30,31,32]. In addition, to overcome this limitation, multiple regression analysis was performed with adjustment variables. As a result, the current oral examination found that subjects with dental caries had a significantly higher DMFT index in all sex types, which may explain some of the limitations.

Second, the cause–effect relationship between osteoporosis and a high DMFT index could not be assessed due to the cross-sectional design of the study. Further longitudinal studies should be performed to help reveal causality.

Third, the relatively low numbers of osteoporosis cases among the pre-menopausal women provided lower statistical power for this group in the multivariable analysis. In KNHANES, each case is associated with a weighting, which means that even a small number of cases may be representative of the nationwide situation, and a survey regression analysis should have been appropriate for the calculation of estimates and their SEs in the present study.

Fourth, variables related to sugar intake were not utilized. The reason is that investigations related to sugar intake have been conducted since 2012, but BMD measurements were conducted only until 2011. However, we have tried to include a number of factors that can affect the dependent variable.

The study also had some strengths. Data for a large cohort of adults over the age of 19 were analyzed according to sex and menopausal status, which controls for the effect of sex and hormonal status on bone health. These data from KNHANES should be representative of the entire Korean population.

## 5. Conclusions

We have shown an association between bone health and oral health in the Korean population. Therefore, the prevention of osteoporosis should be implemented alongside proper oral care, dental caries treatment. However, due to the relative lack of research in this area and the lack of a confirmed cause–effect relationship, it will be necessary to perform a long-term prospective study of this relationship in the future.

## Figures and Tables

**Table 1:** Mean of DMFT index according to the general characteristics of the participants.

	Men			Pre-Menopausal Women			Post-Menopausal Women		
Variables	N (%)	DMFT M (SE)	*p* Value	N(%)	DMFT M (SE)	*p* Value	N (%)	DMFT M (SE)	*p* Value
Age, years			<0.0001			<0.0001			<0.0001
19–29	918 (12.1)	5.8 (0.1)		1152 (22.5)	6.8 (0.1)		-	-	
30–39	1505 (19.7)	4.9 (0.1)		1975 (38.5)	6.5 (0.1)		9 (0.2)	4.2 (1.3)	
40–49	1515 (19.9)	4.5 (0.1)		1695 (33.0)	6.4 (0.1)		180 (4.1)	7.0 (0.3)	
50–59	1362 (17.9)	5.1 (0.1)		309 (6.0)	6.8 (0.3)		1407 (32.0)	7.3 (0.1)	
60–69	1306 (17.2)	7.2 (0.2)		-	-		1487 (33.8)	8.7 (0.1)	
≥70	1008 (13.2)	10.1 (0.2)		-	-		1313 (29.9)	11.5 (0.2)	
Education level			<0.0001			<0.0001			<0.0001
≤Elementary school	1423 (18.7)	7.7 (0.2)		233 (4.5)	6.6 (0.3)		2907 (66.1)	9.6 (0.1)	
Middle school	966 (12.7)	5.8 (0.1)		353 (6.9)	6.1 (0.2)		614 (14.0)	7.3 (0.2)	
High school	2697 (35.4)	5.1 (0.1)		2423 (47.2)	6.3 (0.1)		665 (15.1)	7.7 (0.2)	
≥College	2528 (33.2)	5.3 (0.1)		2122 (41.4)	7.0 (0.1)		210 (4.8)	7.3 (0.3)	
Household Income			<0.0001			<0.0001			<0.0001
Q1 (lowest)	1391 (18.3)	7.0 (0.1)		410 (8.0)	6.5 (0.2)		1658 (37.7)	9.9 (0.2)	
Q2	1928 (25.3)	5.6 (0.1)		1257 (24.5)	6.6 (0.1)		1123 (25.6)	8.7 (0.2)	
Q3	2180 (28.6)	5.2 (0.1)		1744 (34.0)	6.4 (0.1)		831 (18.9)	7.8 (0.2)	
Q4 (highest)	2115 (27.8)	5.3 (0.1)		1720 (33.5)	6.8 (0.1)		784 (17.8)	8.0 (0.2)	
Smoking status			<0.0001			<0.0001			<0.0001
None	1392 (18.3)	5.3 (0.1)		4421 (86.2)	6.6 (0.1)		4011 (91.3)	8.7 (0.1)	
Former	2916 (38.3)	5.9 (0.1)		342 (6.7)	7.0 (0.2)		188 (4.3)	10.1 (0.6)	
Current	3304 (43.4)	5.5 (0.1)		366 (7.1)	5.9 (0.2)		195 (4.4)	9.6 (0.5)	
Drinking experiences			<0.0001			00.2945			<0.0001
No	406 (5.3)	6.7 (0.3)		444 (8.7)	6.7 (0.2)		1583 (36.0)	9.5 (0.2)	
Yes	7206 (94.7)	5.5 (0.1)		4685 (91.3)	6.6 (0.1)		2811 (64.0)	8.4 (0.1)	

DMFT index: DMFT Index is the sum of the number of caries experienced on permanent teeth, the number of teeth lost due to caries experience, and the number of caries on permanent teeth treated. It is a discrete variable from 0 to 28 considering the maximum number of teeth.

**Table 2:** Comparison of DMFT index on bone mineral density by sex and menopausal status.

	Men			Pre-Menopausal Women			Post-Menopausal Women		
	N (%)	DMFT M (SE)	*p* for Trend	N (%)	DMFT M (SE)	*p* for Trend	N (%)	DMFT M (SE)	*p* for Trend
BMD									
Normal	4350 (57.1)	5.3 (0.1)	<0.0001	3342 (65.1)	6.6 (0.1)	0.3715	608 (13.8)	7.2 (0.2)	<0.0001
Osteopenia	2893 (38.0)	5.8 (0.1)		1719 (33.5)	6.6 (0.1)		2127 (48.4)	8.2 (0.1)	
Osteoporosis	371 (4.87)	8.1 (0.3)		70 (1.4)	6.3 (0.5)		1661 (37.8)	10.3 (0.2)	

−2.5 < T-score < −1.0 was regarded as osteopenia. T-score ≤ 2.5 was regarded as osteoporosis.

**Table 3:** The results of multiple survey regression analysis with comparison of DMFT index on bone mineral density by sex and menopausal status.

	Men		Pre-Menopausal Women		Post-Menopausal Women	
	Estimate (95% CI)	*p* Value	Estimate (95% CI)	*p* Value	Estimate (95% CI)	*p* Value
BMD						
Normal	Ref.		Ref.		Ref.	
Osteopenia	0.10 (0.00, 0.20)	0.0447	−0.08 (−0.18, 0.02)	0.1061	0.26 (0.10, 0.43)	0.0018
Osteoporosis	0.98 (0.71, 1.25)	<0.0001	−0.29 (−0.76, 0.17)	0.2166	0.86 (0.63, 1.09)	<0.0001
Age, years						
19–29	Ref.		Ref.		Ref.	
30–39	−0.97 (−1.11, −0.83)	<0.0001	−0.25 (−0.37, −0.14)	<0.0001	0	
40–49	−1.31 (−1.44, −1.18)	<0.0001	−0.10 (−0.22, 0.02)	0.1037	2.95 (2.69, 3.21)	<0.0001
50–59	−0.78 (−0.93, −0.62)	<0.0001	0.40 (0.08, 0.72)	0.0153	3.13 (2.95, 3.31)	<0.0001
60–69	1.17 (0.95, 1.40)	<0.0001	0		4.16 (3.94, 4.37)	<0.0001
≥70	3.67 (3.41, 3.93)	<0.0001	0		6.41 (6.08, 6.73)	<0.0001
Household Income						
Q1(lowest)	−0.12 (−0.28, 0.04)	0.1479	−0.15 (−0.34, 0.04)	0.1167	0.12 (−0.14, 0.39)	0.3604
Q2	−0.12 (−0.25, 0.01)	0.0729	−0.03 (−0.18, 0.12)	0.6944	0.16 (−0.08, 0.40)	0.185
Q3	−0.14 (−0.27, −0.01)	0.0356	−0.26 (−0.34, 0.04)	0.0003	−0.42 (−0.68, −0.16)	0.0015
Q4(highest)	Ref.		Ref.		Ref.	
Education level						
≤Elementary school	0.42 (0.23, 0.61)	<0.0001	−0.47 (−0.70, −0.24)	<0.0001	0.41 (0.13, 0.69)	0.0044
Middle school	−0.37 (−0.53, −0.21)	<0.0001	−0.83 (−1.06, −0.60)	<0.0001	−0.42 (−0.66, −0.18)	0.0007
High school	−0.52 (−0.62, −0.42)	<0.0001	−0.73 (−0.83, −0.62)	<0.0001	0.16 (−0.09, 0.40)	0.2036
≥College	Ref.		Ref.		Ref.	
Smoking status						
None	Ref.		Ref.		Ref.	
Former	0.33 (0.21, 0.45)	<0.0001	0.35 (0.17, 0.52)	0.0001	0.74 (0.15, 1.33)	0.0013
Current	0.41 (0.29, 0.52)	<0.0001	−0.65 (−0.82, −0.49)	<0.0001	0.64 (0.25, 1.03)	0.0147
Drinking experiences						
No	0.49 (0.26, 0.72)	<0.0001	0.10 (−0.12, 0.33)	0.3698	0.22 (0.05, 0.38)	0.0101
Yes	Ref.		Ref.		Ref.	
Periodontal disease						
No	0.30 (0.20, 0.40)	<0.0001	0.28 (0.10, 0.46)	0.0022	0.88 (0.71, 1.05)	<0.0001
Yes	Ref.		Ref.		Ref	
Frequency of toothbrushing per day						
0	−0.47 (−0.71, −0.24)	<0.0001	−0.96 (−1.16, −0.75)	<0.0001	0.40 (−0.12, 0.92)	0.1279
1	−0.56 (−0.76, −0.36)	<0.0001	−0.67 (−0.92, −0.42)	<0.0001	0.15 (−0.34, 0.64)	0.5469
2	−0.39 (−0.58, −0.20)	<0.0001	−0.63 (−0.82, −0.44)	<0.0001	0.11 (−0.31, 0.52)	0.6081
3	−0.23 (−0.43, −0.04)	0.0202	−0.27 (−0.47, −0.06)	0.0097	0.24 (−0.19, 0.66)	0.2757
4	Ref.		Ref.		Ref.	
Necessity of oral treatment						
No	Ref.		Ref.		Ref.	
Yes	1.63 (1.54, 1.72)	<0.0001	0.86 (0.73, 1.00)	<0.0001	0.68 (0.53, 0.84)	<0.0001
Diabetes						
No	Ref.		Ref.		Ref.	
Yes	0.48 (0.29, 0.68)	<0.0001	0.69 (0.27, 1.10)	0.0012	0.08 (−0.13,0.30)	0.4502

## Data Availability

The data supporting the findings of this study are available from The Korea National Health and Nutrition Examination Survey (KNHANES) (https://knhanes.cdc.go.kr https://knhanes.kdca.go.kr/ Accessed 5 June 2022).

## Supplementary Materials

The following supporting information can be downloaded at: https://www.mdpi.com/article/10.3390/ijerph19116917/s1, Table S1: Multiple regression with comparison of DMFT index on bone mineral density by men and post-menopausal women’s age.
