# Supplementary material for: Relationship between Bone Mineral Density and Dental Caries in Koreans by Sex and Menopausal State
Source: Int J Environ Res Public Health. 2022 Jun 5;19(11):6917. doi: 10.3390/ijerph19116917 (PMC9180879; doi:10.3390/ijerph19116917)
Supplement: Supplementary file 1 [file ijerph-19-06917-s001.zip › ijerph-1739928-supplementary.pdf]

Supplementary Tables

Table S1. Multiple regression with comparison of DMFT Index on Bone Mineral Density by Men and Post-menopausal women's age

	Men (<50)		Men (≥50)		Post-menopausal women (<60)		Post-menopausal women (≥60)	
	Estimate (95% CI)	*p* value	Estimate (95% CI)	*p* value	Estimate (95% CI)	*p* value	Estimate (95% CI)	*p* value
BMD								
Normal	Ref.		Ref.		Ref.		Ref.	
Osteopenia	-0.19 (-0.31, -0.08)	0.0012	0.67 (0.51, 0.84)	<.0001	0.35 (0.11, 0.60)	0.0044	0.69 (0.31, 1.06)	0.0003
Osteoporosis	0.61 (0.11, 1.10)	0.0176	1.94 (1.67, 2.21)	<.0001	0.65 (0.32, 0.99)	0.0002	1.80 (1.36, 2.23)	<.0001
House Income								
Q1	-0.44 (-0.61, -0.28)	<.0001	1.74 (1.47, 2.01)	<.0001	-0.46 (-0.76, -0.15)	0.0032	0.65 (0.25, 1.06)	0.0018
Q2	-0.02 (-0.17, 0.13)	0.8094	0.28 (0.06, 0.50)	0.0125	-0.19 (-0.44, 0.06)	0.1294	0.44 (0.01, 0.87)	0.0467
Q3	-0.21 (-0.35, -0.06)	0.0059	0.21 (-0.02, 0.44)	0.0767	-0.63 (-0.88, -0.38)	<.0001	-0.17 (-0.67, 0.33)	0.5054
Q4	Ref.		Ref.		Ref.		Ref.	
Education level								
≤ Elementary school	-1.00 (-1.23, -0.78)	<.0001	1.09 (0.83, 1.35)	<.0001	0.23 (-0.05, 0.50)	0.1115	1.69 (1.43, 1.94)	<.0001
Middle school	-0.87 (-1.05, -0.69)	<.0001	-0.16 (-0.40, 0.07)	0.1603	-0.79 (-1.07, -0.51)	<.0001	1.00 (0.70, 1.30)	<.0001
High school	-0.27 (-0.40, -0.15)	<.0001	-0.74 (-0.94, -0.54)	<.0001	0.13 (-0.14, 0.40)	0.3362	0.56 (0.19, 0.92)	0.0029
≥ College	Ref.		Ref.		Ref.		Ref.	
Smoking status								
None	Ref.		Ref.		Ref.		Ref.	
Former	-0.09 (-0.24, 0.07)	0.2618	0.96 (0.74, 1.17)	<.0001	-0.78 (-1.59, 0.04)	0.0627	1.68 (0.94, 2.42)	<.0001
Current	-0.08 (-0.22, 0.06)	0.2345	0.79 (0.57, 1.02)	<.0001	-0.32 (-0.57, -0.08)	0.0105	1.72 (1.05, 2.40)	<.0001
Drinking experiences								
No	0.38 (-0.02, 0.78)	0.0608	0.99 (0.70, 1.28)	<.0001	-0.06 (-0.36, 0.24)	0.712	0.69 (0.49, 0.88)	<.0001
Yes	Ref.		Ref.		Ref.		Ref.	
Periodontal disease								
No	0.24 (0.13, 0.35)	<.0001	0.74 (0.53, 0.95)	<.0001	-0.11 (-0.38, 0.15)	0.4127	1.59 (1.35, 1.84)	<.0001
Yes	Ref.		Ref.		Ref.		Ref.	
frequency of toothbrushing per day								
0	-0.98 (-1.29, -0.66)	<.0001	0.33 (-0.17, 0.84)	0.1903	0.10 (-0.56, 0.76)	0.769	0.44 (-0.68, 1.56)	0.443
1	-0.77 (-1.02, -0.52)	<.0001	0.07 (-0.36, 0.49)	0.7626	0.27 (-0.09, 0.64)	0.1435	-0.12 (-1.22, 0.98)	0.8302
2	-0.41 (-0.62, -0.19)	0.0002	-0.37 (-0.81, 0.07)	0.1032	0.24 (-0.13, 0.61)	0.2034	-0.34 (-1.36, 0.67)	0.507
3	-0.31 (-0.56, -0.07)	0.0105	0.13 (-0.31, 0.57)	0.5571	0.42 (0.04, 0.80)	0.0297	-0.20 (-1.21, 0.81)	0.7027
4	Ref.		Ref.		Ref.		Ref.	

(Continued)

	Men (<50)		Men (≥50)		Post-menopausal women (<60)		Post-menopausal women (≥60)	
	Estimate (95% CI)	p value	Estimate (95% CI)	p value	Estimate (95% CI)	p value	Estimate (95% CI)	p value
Necessity of oral treatment								
No	Ref.		Ref.		Ref.		Ref.	
Yes	1.93 (1.80, 2.05)	<.0001	1.11 (0.98, 1.25)	<.0001	1.06 (0.90, 1.23)	<.0001	0.41 (0.16, 0.65)	0.0011
Diabetes								
No	Ref.		Ref.		Ref.		Ref.	
Yes	-0.42 (-0.73, -0.11)	0.0081	0.77 (0.54, 1.00)	<.0001	0.34 (-0.07, 0.76)	0.102	0.05 (-0.22, 0.31)	0.7278
